# Evaluation of biosecurity measures to prevent indirect transmission of porcine epidemic diarrhea virus

**DOI:** 10.1186/s12917-017-1017-4

**Published:** 2017-04-05

**Authors:** Yonghyan Kim, My Yang, Sagar M. Goyal, Maxim C-J. Cheeran, Montserrat Torremorell

**Affiliations:** Department of Veterinary Population Medicine, College of Veterinary Medicine, University of Minnesota, 1988 Fitch Ave, St. Paul, MN 55108 USA

**Keywords:** Porcine epidemic diarrhea virus, Indirect transmission, Farm personnel, Animal movement, Biosecurity, Fomites

## Abstract

**Background:**

The effectiveness of biosecurity methods to mitigate the transmission of porcine epidemic diarrhea virus (PEDV) via farm personnel or contaminated fomites is poorly understood. This study was undertaken to evaluate the effectiveness of biosecurity procedures directed at minimizing transmission via personnel following different biosecurity protocols using a controlled experimental setting.

**Results:**

PEDV RNA was detected from rectal swabs of experimentally infected (INF) and sentinel pigs by real-time reverse transcription polymerase chain reaction (rRT-PCR). Virus shedding in INF pigs peaked at 1 day post infection (dpi) and viral RNA levels remained elevated through 19 dpi. Sentinel pigs in the low biosecurity group (LB) became PEDV positive after the first movement of study personnel from the INF group. However, rectal swabs from pigs in the medium biosecurity (MB) and high biosecurity (HB) groups were negative during the 10 consecutive days of movements and remained negative through 24 days post movement (dpm) when the first trial was terminated.

Viral RNA was detected at 1 dpm through 3 dpm from the personal protective equipment (PPE) of LB personnel. In addition, at 1 dpm, 2 hair/face swabs from MB personnel were positive; however, transmission of virus was not detected. All swabs of fomite from the HB study personnel were negative.

**Conclusions:**

These results indicate that indirect PEDV transmission through contaminated PPE occurs rapidly (within 24 h) under modeled conditions. Biosecurity procedures such as changing PPE, washing exposed skin areas, or taking a shower are recommended for pig production systems and appear to be an effective option for lowering the risk of PEDV transmission between groups of pigs.

## Background

Porcine epidemic diarrhea (PED) is a highly contagious viral disease that causes severe diarrhea in pigs [1]. In May 2013, the porcine epidemic diarrhea virus (PEDV) was first reported in the US, causing significant economic losses to the swine industry due to high mortality rates in piglets. Over 8 million pigs died due to PEDV, leading to an estimated total industry economic loss of more than 1.8 billion US dollars [2]. PEDV is an enveloped, single-stranded, positive-sense RNA virus belonging to the *Coronaviridae* family in the genus *Alphacoronavirus* [1, 3]. The virus is shed in feces of infected pigs and transmitted via the fecal-oral route. PEDV can be transmitted either by direct contact between infected and susceptible pigs or indirectly through contaminated fomites. Transmission via pig transportation has been reported as a major risk factor for the spread of PEDV [4, 5]. Five percent of PEDV negative trailers became contaminated during the unloading process at slaughterhouse facilities handling infected pigs [4]. Contaminated feed has also been implicated in the spread of PEDV, and both food ingredients (viz. dried spray plasma) and cross-contamination at the feed mill or from other sources have been implicated in the spread of PEDV [6–11]. PEDV has also been detected in air samples and aerosol transmission has been suspected as a potential source of disease transmission in high pig dense areas [12].

In order to mitigate transmission of PEDV within and between farms, producers employ a range of biosecurity practices. These practices include disinfecting footwear and changing clothing of visitors or personnel prior to entering farm premises, washing and sanitizing delivery trucks or vehicles entering the farm, and controlling insects. Controlling transmission via feed can be done by using feed additives such as formaldehyde [13] and transmission via transport can be minimized by implementing proper cleaning and disinfection methods [14]. However, these implementations are not always practical or cost effective. The effectiveness of methods to mitigate transmission via farm personnel or contaminated fomites are less understood given that intervention strategies at the farm level have not been properly investigated. Furthermore, Stevenson et al. indicated that even shower-in/shower-out facilities with excellent biosecurity protocols also reported PEDV outbreaks [15]. Given the limited knowledge available on how biosecurity procedures may disrupt the transmission cycle of PEDV, the present study was undertaken to evaluate the effectiveness of biosecurity procedures directed at minimizing transmission via personnel following different biosecurity protocols using a controlled experimental setting.

## Methods

### Animals and animal housing

Forty-eight, 3-week-old crossbred pigs including both male and females were obtained from a farm with no history of PEDV infection and were housed at the St. Paul animal research isolation units at the University of Minnesota. After arrival (2 days before the start of the study), rectal swabs from all pigs were collected and tested by real-time reverse transcription polymerase chain reaction (rRT-PCR) for PEDV, transmissible gastroenteritis virus (TGEV) and porcine delta coronavirus (PDCoV) at the University of Minnesota Veterinary Diagnostic Laboratory (St. Paul, MN, USA).

**Fig. 1:**
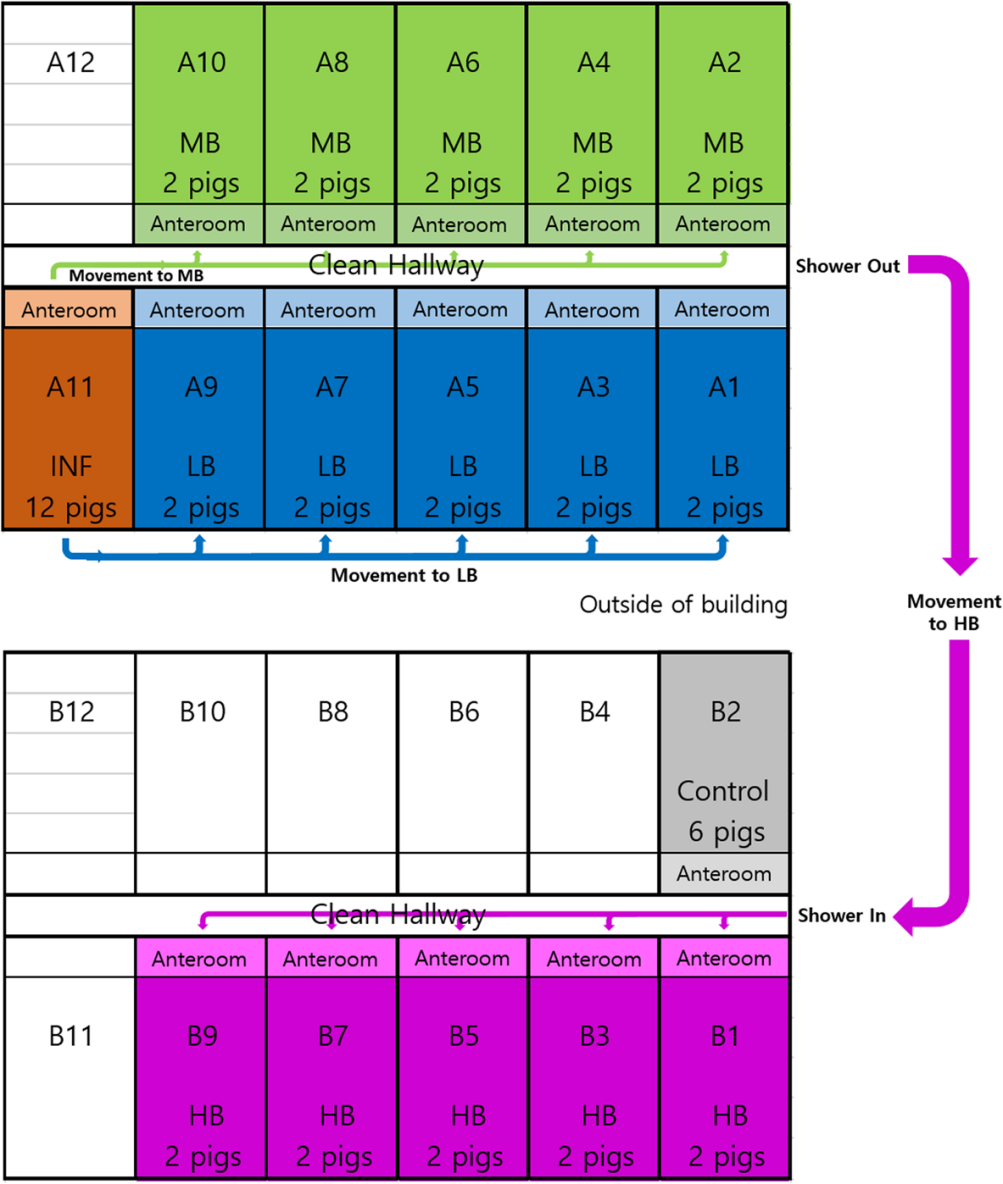
Movement from infected source group (INF) to low biosecurity group (LB) and INF to medium biosecurity group (MB)

The pigs were housed in 17 separate rooms that were independently operated from each other as described below. All individual rooms had anterooms with footbaths, a sink for hand and face washing, a storage area of 2.08 m^2^, and an animal housing area of 7.28 m^2^. Rooms were connected through a clean common hallway as shown in Fig. 1. The floor of the animal housing area was constructed with solid concrete and each animal housing area had a single water line with two water nipples as a source of drinking water. Prior to introducing the pigs into the rooms, environmental swabs were collected from the floors and confirmed PEDV negative by rRT-PCR. Ventilation for all rooms was kept under negative differential pressure to the main corridor, having one air inlet and one exhaust vent per room. The air supply was conditioned with a 3 ply panel filter (TRI-DEK® 15/40, TRI-DIM Filter Corp., Louisa, VA, USA) and 100% of exhaust air was filtered through a HEPA filter (XH Absolute HEPA filter, Camfil, Stockholm, Sweden).

The pigs were randomly distributed as described below and treated with a single intramuscular dose of enrofloxacin (0.5 mL/pig; Baytril®, Bayer HealthCare AG, Leverkusen, Germany) to control respiratory disease associated with Haemophilus parasuis 1 day prior to infection. In the first trial of the study, forty eight 3-week-old piglets were randomly assigned to 5 experimental groups: (i) 10 pigs were infected with PEDV and 2 contact sentinel pigs were housed together to serve as contact sentinels (INF group); (ii) 10 pigs (5 replicates of 2 each) were assigned to low biosecurity (LB) sentinel groups; (iii) 10 pigs (5 replicates of 2 each) were assigned to medium biosecurity (MB) sentinel groups; (iv) 10 pigs (5 replicates of 2 each) were assigned to high biosecurity (HB) sentinel groups and (v) six pigs were assigned to a negative control (NC) group, which were uninfected and handled separately. Trial 1 lasted a total of 27 days.

In the 2nd trial of the study, twenty-three 6-week-old pigs were assigned to 4 different groups: (i) 3 pigs were infected with PEDV and 1 contact sentinel pig were assigned to the INF group; (ii) 4 pigs (1 replicate) were assigned to LB sentinel group; (iii) 12 pigs (3 replicates of 4 each) were assigned to MB sentinel groups; and (iv) 3 pigs (1 replicate) were assigned to NC group. Trial 2 lasted a total of 13 days.

### Study personnel

Study personnel were exclusively assigned to handle pigs in this study and had no direct contact with other pigs or with PEDV-infected pigs from another source for the entire duration of this study. Personnel entering LB, MB, and HB sentinel rooms had direct contact with infected pigs in the INF room only and performed all necessary procedures (e.g. pig fecal swab collection, pig blood collection, feeding of pigs, cleaning of room) in their designated rooms as assigned during the deputed sampling and movement days.

### Clothing and personal protective equipment

All study personnel showered in the research animal facility prior to donning the facility-dedicated clothing and personal protective equipment (PPE). After showering, personnel put on clean scrubs, a pair of disposable plastic boots and entered the animal isolation corridor after stepping through an iodine footbath. In the animal isolation clean hallway, personnel put on disposable Tyvek® coverall (DuPont, Wilmington, DE, USA), nitrile gloves, and a bouffant cap for the first trial of the study. In the 2nd trial, a cloth coverall was used instead of Tyvek® coverall. Upon entry into the anterooms through another iodine footbath, personnel put on face shield and room-specific rubber boots. Before entering the animal housing area, personnel put on another pair of disposable plastic boots over their rubber boots.

## Experimental design

### Infected group (INF)

For the first trial of the study, ten PEDV negative 3-week-old pigs were inoculated with PEDV (USA/Colorado/2013) strain of passage 16 via the intra-gastric route. Each pig was infected with 10 mL of the virus inoculum containing 3.6×10^4^ 50% tissue culture infective dose (TCID_50_) per mL. Two uninfected PEDV negative 3-week-old pigs were housed with infected animals to serve as sentinels to assess transmission by direct contact. During the 2nd trial, which was performed after the 1st, three PEDV negative 6-week-old pigs were inoculated with gastrointestinal mucosal scrapings obtained from animals infected with the PEDV virulent strain, by intra-gastric route. An uninfected 6-week-old pig was added to this group as a direct contact sentinel. All study personnel interacted with infected pigs for the movements.

### Movement between experimental groups

A movement was defined as the process when study personnel moved from the INF room to either the LB, MB, or HB sentinel rooms (Table 1). The first movement started approximately 44 h following the experimental inoculation of pigs in the INF group at a time when direct contact transmission was known to have occurred.

**Table 1:** Summary of experimental design and biosecurity procedures followed prior to entry into the low, medium or high biosecurity rooms and the negative control room

From	To	Procedures	Trial 1	Trial 2
INFECTED (INF)	Low Biosecurity (LB)	Direct movement from INF to LB through soiled corridor	10 pigs	4 pigs
		No change of clothes or footwear between INF and LB	2 pigs/room	4 pigs/room
		No washing of hands or face	5 replicates	1 replicate
	Medium Biosecurity (MB)	Movement from INF to MB through clean corridor only after procedures were followed	10 pigs	12 pigs
		Wash hands and face	2 pigs/room	4 pigs/room
		Change clothes and footwear	5 replicates	3 replicates
	High Biosecurity (HB)	Movement from INF to HB through clean corridor only after procedures were followed	10 pigs	NA
		Shower	2 pigs/room	NA
		Change clothes and footwear	5 replicates	NA
	Negative control (NC)	No movement of people or fomities between INF. LB, MB or HB and negative control	6 pigs	3 pigs
		Dedicated study personnel different from personnel attending the other groups	6 pigs/room	3 pigs/room
		Shower, clean clothes and footwear each time entering the room	1 replicate	1 replicate

#### Exposure of study personnel to INF pigs

All study personnel who participated in movements between experimental groups were in contact first with pigs in the INF group for 45 min. Personnel interacted directly with the pigs by handling the pigs, collecting samples from them, and allowing pigs to come in contact with personnel clothes and PPE, e.g. biting, sniffing, and rubbing. Accordingly, potential infectious secretions and feces could be transferred to clothing and PPE worn by the study personnel.

#### Movement from infected room to LB rooms

Following the interaction period with pigs in the INF group, study personnel who were designated to LB rooms placed their used nitrile gloves, disposable plastic boots, bouffant cap and coveralls into a clean plastic bag while in the storage area of the INF room. LB room study personnel exited INF room through a soiled (outside) corridor and entered directly into the LB sentinel holding room through an exit door in the soiled corridor, without stepping into iodine footbaths (Fig. 1). The LB room study personnel re-donned their used PPE, including nitrile gloves, disposable plastic boots, bouffant cap and coveralls, in the LB anteroom area. Prior to initiating contact with LB sentinel pigs, each person collected four separate swab samples from their (i) used coveralls, (ii) used disposable plastic boots, (iii) used nitrile gloves, and (iv) used bouffant cap and face/hair area in the LB storage area. After collecting the swab samples, personnel collected rectal swab samples from LB room sentinel pigs and interacted with LB room sentinel pigs for 45 min as previously described. All study personnel designated to LB rooms did not wash their hands and face prior to contact with LB sentinel pigs. These movements were scheduled once a day for 9 more consecutive days and terminated after LB sentinel pigs tested positive for PEDV.

#### Movement from infected room to MB rooms

Following interaction with INF pigs, study personnel collected four separate swabs from the surface of used (i) coveralls, (ii) disposable plastic boots, (iii) nitrile gloves, and (iv) bouffant caps and face/hair area in the INF room storage area. All MB room study personnel exited the INF room through the anteroom and removed their used coveralls, disposable plastic boots, latex gloves, and bouffant cap, and washed their hands and face with soap and water for approximately 20–40 s according to the Centers for Disease Control and Prevention (CDC) guidelines [18] prior to exiting the room into the clean corridor (Fig. 1). In the clean hallway, study personnel donned new coveralls and bouffant cap and collected four separate fomite swab samples from the new PPE, including (i) coveralls, (ii) disposable plastic boots, and from their (iii) hands, and (iv) bouffant cap and hair/face area prior to entering the anteroom of MB sentinel rooms. Here, study personnel washed again their hands and face and then, put on gloves, protective eyewear and room-specific rubber boots. Before entering the MB room animal housing area, personnel put on another pair of disposable plastic boots over their rubber boots. MB study personnel collected rectal swab samples from sentinel pigs and interacted with them, as described above. These movements were completed once a day over nine more consecutive days.

#### Movement from infected room to HB rooms

The HB animals were housed in a separate building located approximately 10 m away. After interacting with pigs in the LB or MB treatment group, study personnel showered with soap and shampoo for approximately 10 min before donning a new set of facility-dedicated scrubs and a pair of new disposable plastic boots. Study personnel donned new PPE, interacted again with pigs in the INF group, and took a full shower again before donning a new set of facility-dedicated scrubs and a pair of new disposable plastic boots before entering the isolation unit where the HB animals were housed. Study personnel entered the animal isolation hallway through an iodine footbath, then donned new coveralls and bouffant cap. Each of the study personnel collected four separate fomite swab samples from the new PPE, hands, and bouffant cap and hair/face area as described above. In the anteroom, study personnel washed their hands and face again, donned gloves, protective eyewear, and room-specific rubber boots with disposable plastic boots over them before entering the animal housing area. All study personnel collected rectal swab samples from HB room sentinel pigs and interacted with them as described above. These movements were completed twice a day over nine more consecutive days.

### Collection of rectal and fomite swabs

Fomite and rectal swab samples were collected using a sterile rayon-tipped swab (BD CultureSwab™, liquid Stuart medium, single plastic applicator, Becton Dickinson and Co., Sparks, MD, USA). Fomite swabs were collected from coveralls, disposable plastic boots, hands or nitrile gloves, bouffant cap, face and hair areas using a zigzag pattern to cover maximum surface area prior to interacting with pigs in each biosecurity group. Rectal swabs were collected daily. Following collection, each swab was suspended in 2 mL transport media solution of Dulbecco’s minimal essential medium (Gibco® DMEM, Thermo Fisher Scientific Inc., Waltham, MA, USA) containing 2% Bovine Albumin Fraction V 7.5% solution (Gibco® BSA, Thermo Fisher Scientific Inc., Waltham, MA, USA), 1% Antibiotic-Antimycotic, 100× solution (Gibco® Anti-Anti, Thermo Fisher Scientific Inc., Waltham, MA, USA), 0.15% Trypsin-TPCK, 1 mg/mL (Sigma-Aldrich, St. Louis, MO, USA) and 0.1% Gentamicin-Sulfate, 50 mg/mL (Lonza Inc., Walkersville, MD, USA). An aliquot (50 μL) of the swab suspension sample was used to extract RNA for rRT-PCR, and the remainder of the samples were stored at −80 °C. Swab samples were tested for the presence of PEDV Spike (S) gene by rRT-PCR. Briefly, RNA was extracted from eluent using the MagMAX™-96 Viral RNA Isolation Kit (ThermoFisher Scientific, Waltham, MA, USA), according to the manufacturer’s instructions. A primer pair was designed to amplify a portion of the PEDV S gene with the following sequences: Forward 1910: ACGTCCCTTTACTTTCAATTCACA and Reverse 2012: TATACTTGGTACACACATCCAGAGTCA. PCR amplification was quantified using a FAM labeled probe 1939: FAM-TGAGTTGATTACTGGCACGCCTAAACCAC-BHQ. The primers and hydrolysis probe set were added to the AgPath-ID™ One-Step RT-PCR Reagents (ThermoFisher Scientific, Waltham, MA, USA) with 5 μl of extracted total RNA and amplified with the ABI 7500 Fast Real-Time PCR System (Thermo Fisher Scientific, Waltham, MA, USA) using the following condition: reverse transcription at 48 °C for 10 min; denaturation at 95 °C for 10 min; 45 cycles of denaturation at 95 °C for 15 s and annealing at 60 °C for 45 s.

## Results

### Fecal shedding

**Fig. 2:**
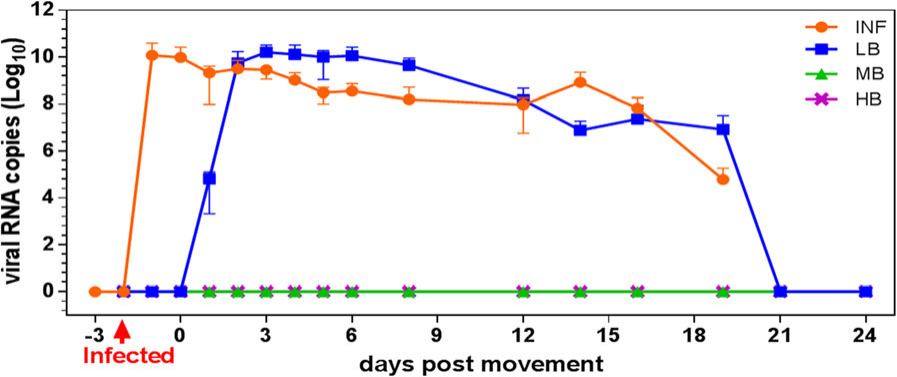
Viral shedding of pigs (1st trial). Movements were terminated at 10 dpi. Data presented are average values of viral RNA copies (± SD) of infected source group (INF) (*n* = 12), low biosecurity group (LB) (*n* = 10), medium biosecurity group (MB) (*n* = 10), and high biosecurity group (HB) (*n* = 10) groups

**Fig. 3:**
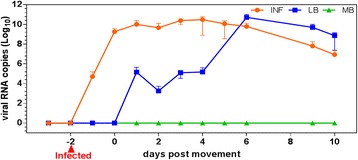
Viral shedding of pigs (2nd trial). Movements were terminated at 10 dpi. Data presented are average values of viral RNA copies (± SD) of infected source group (INF) (*n* = 4), low biosecurity group (LB) (*n* = 4), and medium biosecurity group (MB) (*n* = 12)

In both studies, pigs had limited clinical signs of diarrhea. Diarrhea was mild and transient in about half of the pigs. In both studies, PEDV RNA was detected by rRT-PCR from rectal swabs of pigs in the INF group at 1-day post infection (dpi), indicative of virus shedding from inoculated pigs. Rectal swabs of direct contact sentinel pigs, co-housed with the INF group in both trials, tested rRT-PCR positive at 2 dpi (Tables 2 and 3), 1 day after virus was detected in inoculated pigs. Virus shedding in pigs of the INF group, measured as viral RNA copies per rectal swab, peaked at 1 dpi and viral RNA levels remained elevated through 19 dpi (Fig. 2). During the 2nd trial, rectal swabs of INF pigs remained positive until 12 dpm when that experiment was terminated (Fig. 3).

**Table 2:** Number of porcine epidemic diarrhea virus positive pigs (1st trial)

Days Post Infection	−1	0	1	2	3	4	5	6	7	8	9	10	11	12
Infection group	0/10	0/10	10/10	10/10	10/10	10/10	10/10	10/10	10/10	10/10	10/10	10/10	10/10	10/10
Infection group sentinel	0/2	0/2	0/2	2/2	2/2	2/2	2/2	2/2	2/2	2/2	2/2	2/2	2/2	2/2
Days After Movement				0	1	2	3	4	5	6	7	8	9	10
Low biosecurity				0/10	9/10	10/10	10/10	10/10	10/10	10/10	10/10	10/10	10/10	10/10
Medium biosecurity				0/10	0/10	0/10	0/10	0/10	0/10	0/10	0/10	0/10	0/10	0/10
High biosecurity				0/10	0/10	0/10	0/10	0/10	0/10	0/10	0/10	0/10	0/10	0/10

**Table 3:** Number of porcine epidemic diarrhea virus positive pigs (2nd trial)

Days Post Infection	−1	0	1	2	3	4	5	6	7	8	9	10	11	12
Infection group	0/3	0/3	3/3	3/3	3/3	3/3	3/3	3/3	3/3	3/3	3/3	3/3	3/3	3/3
Infection group sentinel	0/1	0/1	0/1	1/1	1/1	1/1	1/1	1/1	1/1	1/1	1/1	1/1	1/1	1/1
Days After Movement				0	1	2	3	4	5	6	7	8	9	10
Low biosecurity				0/4	4/4	4/4	4/4	4/4	4/4	4/4	4/4	4/4	4/4	4/4
Medium biosecurity				0/12	0/12	0/12	0/12	0/12	0/12	0/12	0/12	0/12	0/12	0/12

Movements were started at 2 dpi of the INF group. Sentinel pigs in the LB group tested PEDV positive on rectal swabs 24 h after the first movement. Viral RNA was detected in 10 out of 10 sentinel pigs during the 1st trial and 4 out of 4 sentinel pigs in the 2nd trial (Tables 2 and 3). Viral shedding in the LB group of 1st trial was undetectable after 21 dpm (Fig. 2). Rectal swabs from pigs in the MB and HB groups tested rRT-PCR negative during the 10 consecutive days of movement and remained negative through 24 dpm, when the first trial was terminated. Rectal swabs from pigs in the NC group remained negative for the entire duration of the study.

### Fomite swabs

Fomite swab samples collected on 1, 2, and 3 dpm from hair/face, hands, coverall, and boots prior to contact with each group of sentinel pigs were tested by rRT-PCR to determine where PEDV was carried on each person that could potentially contribute to virus transmission (Table 4). Viral RNA was detected at 1 dpm through 3 dpm from all LB group PPE fomite swab samples during the first trial. PEDV was detected in one coverall swab in the 2nd study at 1 dpm and in all PPE at 2 and 3 dpm. In addition, at 1 dpm, 2 hair/face swabs from MB personnel were positive in the 1st study, even though transmission of virus was not detected. All fomite swabs from HB study personnel tested negative.

**Table 4:** Number of porcine epidemic diarrhea virus positive fomite swabs prior to contact with pigs in the respective groups and mean (±SD) cycle threshold RT-PCR values for positive samples

Group	Swab	Movement day					
		1st study			2nd study		
		1	2	3	1	2	3
Negative	Bouffant cap, hair, face area	(0/5)^a^	(0/5)	(0/5)	(0/4)	(0/4)	(0/4)
	Coverall	(0/5)	(0/5)	(0/5)	(0/4)	(0/4)	(0/4)
	Hands	(0/5)	(0/5)	(0/5)	(0/4)	(0/4)	(0/4)
	Boots	(0/5)	(0/5)	(0/5)	(0/4)	(0/4)	(0/4)
LB	Bouffant cap, hair, face area	(3/5) (31.58 ± 1.03) ^b^	(2/5) (33.62 ± 0.16)	(5/5) (32.66 ± 1.58)	(0/1)	(1/1) (31.62)	(1/1) (33.58)
	Coverall	(5/5) (26.16 ± 3.17)	(5/5) (29.28 ± 2.22)	(5/5) (27.96 ± 3.96)	(1/1) (33.40)	(1/1) (29.27)	(1/1) (24.60)
	Used gloves	(5/5) (28.81 ± 3.83)	(4/5) (28.01 ± 2.98)	(5/5) (28.76 ± 2.21)	(0/1)	(1/1) (28.03)	(1/1) (30.01)
	Boots	(5/5) (26.30 ± 4.44)	(5/5) (27.42 ± 6.22)	(5/5) (24.51 ± 3.94)	(0/1)	(1/1) (28.74)	(1/1) (28.54)
MB	Bouffant cap, hair, face area	(2/5) (30.75 ± 0.93)	(0/5)	(0/5)	(0/3)	(0/3)	(0/3)
	Coverall	(0/5)	(0/5)	(0/5)	(0/3)	(0/3)	(0/3)
	Hands	(0/5)	(0/5)	(0/5)	(0/3)	(0/3)	(0/3)
	Boots	(0/5)	(0/5)	(0/5)	(0/3)	(0/3)	(0/3)
HB	Bouffant cap, hair, face area	(0/5)	(0/5)	(0/5)			
	Coverall	(0/5)	(0/5)	(0/5)			
	Hands	(0/5)	(0/5)	(0/5)			
	Boots	(0/5)	(0/5)	(0/5)			

^a^ Number of positive

^b^ Ct value (avg. ± S.D)

## Discussion

Although several aspects of PEDV transmission have been examined, the efficiency by which biosecurity measures prevent indirect transmission of PEDV has been largely unexplored. In the current study, we sought to address this by modifying biosecurity measures in a controlled experimental design, using study personnel to simulate movements between rooms that reflect situations within swine farms around the country. Graded biosecurity stringency was designed into movements made by study personnel between a known infected room and sentinel rooms. As expected, direct-contact sentinel pigs showed signs of PEDV infection 24 h after viral shedding was detected in infected pigs supporting the view that PEDV is highly contagious [16]. Movements between INF and sentinel rooms were designed to begin when viral shedding peaked in the source group at 2 dpi. Movements to the LB rooms simulated indirect transmission in the absence of biosecurity protocols. Interestingly, transmission to the LB sentinel groups happened surprisingly rapidly. Virus shedding in the LB sentinels was detected 24 h after the first movement into the room again providing proof of the contagious nature of PEDV. Samples from PPE of all study personnel in contact with experimentally infected pigs were found to be contaminated with PEDV by rRT-PCR, and transmitted infection to the LB sentinel pigs even though virus infectivity on PPE was not tested. This information is relevant since it helps explain the rapid spread of PEDV within populations even in the absence of direct contact pig transmission.

Among the graded biosecurity measures designed to break the virus transmission cycle, movements into the MB sentinel groups showed no evidence of transmission even though swabs from MB study personnel’s hair and face were PEDV rRT-PCR positive. Transmission of PEDV with MB protocols may have been limited by low dose of virus, presence of non-infectious virus, inadequate interaction of pigs with contaminated PPE/surfaces, or the decreased efficiency of fecal oral transmission route from these contaminated areas. Similar experiments using an influenza virus transmission model showed a breakdown of medium biosecurity measures after 10 consecutive movements [17]. Swabs from HB group study personnel tested PEDV rRT-PCR negative even on the hair and face. Similarly, HB sentinel pigs were rRT-PCR negative for 10 consecutive days. These results together indicate that taking a shower and changing PPE before contacting pigs is an ideal way to completely prevent indirect viral transmission in conditions generally seen in farms. Although our results also support that only changing PPE and washing skin exposed areas is beneficial to decrease the risk of PEDV transmission, there may still be an inherent risk of PEDV transmission from contaminated body surfaces on personnel. Hence, only changing PPEs might not be the most effective way to protect against spread of PEDV.

Our results also indicate that breaches in biosecurity procedures can very rapidly transmit PEDV to naïve herds through indirect means (viz. contaminated fomites). These findings also suggest that contact with PEDV contaminated fomites for a sufficient time is an efficient source of infection and likely plays a role in the rapid transmission of PEDV when there is adequate contact with fomites. Previous studies have suggested that fomites may be an effective mode of PEDV transmission [6, 7, 9, 11–13, 16]. These previous studies rely on PCR detection of viral RNA particles or demonstration of infectious PEDV in cell culture assays to suggest the possibility of transmission by fomites. For example, airborne transmission [12], vehicles [4], feed [7, 10, 16], storage bags [6], personnel working with pigs [6] and other fomites have tested positive for PEDV indicating their possible role in viral transmission and should be considered as a source for virus spread. However, these studies lack a tangible demonstration of the ability of contaminated fomites to infect pigs, either in an experimental setting or in a farm, except for the role of contaminated feed. The present study provides evidence that personnel exposed to infected pigs can transmit the virus to a naïve population, when basic biosecurity procedures are not followed.

The experimental design in the present studies allowed a 45-min contact time with animals under the assumption that most routine activities in a farm, based on the size of the pen and number of pigs housed, may be completed within that time frame. However, one cannot rule out the possibility that transmission may occur with medium biosecurity, if longer interaction periods or larger infected source groups were used in the design. Contact with infected pigs for more than 45 min and/or 10 movements could have increased the probability of PEDV transmission to naïve sentinel pigs. However, we observed that with low biosecurity procedures, transmission and infection of PEDV was both efficient and rapid. This data provides evidence that spread of PEDV within farms may occur efficiently with failures in biosecurity procedures.

Results presented here should be considered carefully as many factors, including contact time, exposure time, viral dose, time after exposure to virus and other experimental conditions may influence the outcome of transmission studies. However, this experimental study highlights the main advantages of good biosecurity procedures in breaking the transmission cycle between rooms. The fact that PEDV transmission occurred under low biosecurity procedures indicated that the virus could spread easily through contaminated fomites worn by personnel. These results provide critical information to develop effective biosecurity procedures and will have potential applications for the development and implementation of transmission control policies in swine production systems. Our results are also relevant to design biosecurity measures to control the spread of other pathogens of similar characteristics and transmission routes than PEDV such as transmissible gastroenteritis virus and porcine deltacoronavirus.

## Conclusions

In conclusion, these results indicate the indirect transmission of PEDV through contaminated personnel PPEs occurs rapidly under modeled conditions and to prevent transmission between groups of pigs, changing PPE and/or taking a shower is recommended as an effective option to lower the risk of virus spread.

## Abbreviations

dpi: Days post infection
dpm: Days post movement
HB: High biosecurity
INF: Infection
LB: Low biosecurity
MB: Medium biosecurity
NC: Negative control
PDCoV: Porcine delta coronavirus
PED: Porcine epidemic diarrhea
PEDV: Porcine epidemic diarrhea virus
PPE: Personal protective equipment
rRT-PCR: Real-time reverse transcription polymerase chain reaction
TCID: Tissue culture infective dose
TGEV: Transmissible gastroenteritis virus
